# Intestine-derived α-synuclein initiates and aggravates pathogenesis of Parkinson’s disease in *Drosophila*

**DOI:** 10.1186/s40035-022-00318-w

**Published:** 2022-10-17

**Authors:** Wei Liu, Kah-Leong Lim, Eng-King Tan

**Affiliations:** 1 School of Plant Protection; Anhui Province Key Laboratory of Crop Integrated Pest Management; Anhui Province Engineering Laboratory for Green Pesticide Development and Application, Anhui Agricultural University, Hefei, 230036 China; 2 Department of Neurology, National Neuroscience Institute, Singapore, 308433 Singapore; 3 Department of Research, National Neuroscience Institute, Singapore, Singapore; 4 Research, Lee Kong Chian School of Medicine, Singapore, Singapore; 5 Neuroscience and Behavioural Disorders Program, Duke-NUS Medical School, Singapore, 169857 Singapore

**Keywords:** Intestine, α-Synuclein, Parkinson’s disease, Microbiome

## Abstract

**Background:**

Aberrant aggregation of α-synuclein (α-syn) is a key pathological feature of Parkinson’s disease (PD), but the precise role of intestinal α-syn in the progression of PD is unclear. In a number of genetic *Drosophila* models of PD, α-syn was frequently ectopically expressed in the neural system to investigate the pathobiology.

**Method:**

We investigated the potential role of intestinal α-syn in PD pathogenesis using a *Drosophila* model. Human α-syn was overexpressed in *Drosophila* guts, and life span, survival, immunofluorescence and climbing were evaluated. Immunofluorescence, Western blotting and reactive oxygen species (ROS) staining were performed to assess the effects of intestinal α-syn on intestinal dysplasia. High‐throughput RNA and 16S rRNA gene sequencing, quantitative RT‐PCR, immunofluorescence, and ROS staining were performed to determine the underlying molecular mechanism.

**Results:**

We found that the intestinal α-syn alone recapitulated many phenotypic and pathological features of PD, including impaired life span, loss of dopaminergic neurons, and progressive motor defects. The intestine-derived α-syn disrupted intestinal homeostasis and accelerated the onset of intestinal ageing. Moreover, intestinal expression of α-syn induced dysbiosis, while microbiome depletion was efficient to restore intestinal homeostasis and ameliorate the progression of PD. Intestinal α-syn triggered ROS, and eventually led to the activation of the dual oxidase (DUOX)–ROS–Jun N-terminal Kinase (JNK) pathway. In addition, α-syn from both the gut and the brain synergized to accelerate the progression of PD.

**Conclusions:**

The intestinal expression of α-syn recapitulates many phenotypic and pathologic features of PD, and induces dysbiosis that aggravates the pathology through the DUOX–ROS–JNK pathway in *Drosophila*. Our findings provide new insights into the role of intestinal α-syn in PD pathophysiology.

**Supplementary Information:**

The online version contains supplementary material available at 10.1186/s40035-022-00318-w.

## Background

Parkinson’s disease (PD) is the second most common debilitating neurodegenerative disorder, with prevalence of 1% in individuals over 60 years old worldwide [1, 2]. PD is clinically characterized by motor deficits, including bradyskinesia, resting tremor, muscle rigidity, and postural instability, and causes significant morbidity as well as decreases in psychosocial health and physical quality of life [3, 4]. A key pathological feature of PD is the selective loss of dopaminergic (DA) neurons in the substantia nigra pars compacta. Due to the limited understanding of the etiology of PD, currently no therapies can reverse or decelerate the progression of PD associated with the demise of DA neurons. PD is a synucleinopathy, characterized by the presence of misfolded α-synuclein (α-syn, encoded by the *SNCA* gene) [5, 6]. α-Syn is constitutively expressed throughout life, but can form protofibrils and fibrils in pathological conditions, becoming a major component of Lewy bodies. Gene multiplications and point mutations in *SNCA* can promote α-syn aggregation, leading to the formation of insoluble amyloid fibrils [7, 8]. Moreover, misfolded α-syn taken up by another neuron acts as a template to misfold other α-syn molecules, causing cell-to-cell spread in a prion-like manner [9, 10]. Clinical and pathological evidence shows that α-syn aggregation originates in the intestine and progresses to the brain. Therefore, Braak posits a hypothesis that pathologic α-syn ascends vagal fibers to the nodose ganglion and brainstem nuclei [11, 12]. Emerging studies have supported this concept by showing that injecting α-syn fibrils into the intestine aggravates the pathology of PD [13, 14]. However, how the intestine-derived α-syn initiates and aggravates neurodegeneration in PD still needs to be further elucidated. The *Drosophila* model allows us to investigate whether the intestine-derived α-syn could recapitulate the disease phenotypes of PD and provide additional pathophysiologic insights.

The pathophysiology of PD involves a variety of factors, including environmental toxins and mutations in specific genes. The intestine harbors a complex microbiome that profoundly impacts a range of host physiologies and diseases [15, 16]. Dysbiosis is sufficient to alter immune surveillance mechanisms, promote intestinal proliferation, and trigger chronic inflammation [17], all of which could play key roles in PD pathogenesis. Emerging studies have revealed that PD patients exhibit altered intestinal microbial composition and function [18–20], raising the possibility that the intestinal microbiota influence PD pathogenesis by regulating synucleinopathies. Indeed, studies have found that the intestinal microbiota can promote α-syn aggregation and the development of motor disturbances in a mouse model of PD [21]. Interestingly, a recent study reported that the curli-producing bacteria increase aggregation of the amyloid protein α-syn in aged rats and nematodes [22], further emphasizing the role of the intestine in PD. However, the mechanism by which intestinal α-syn interacts with the intestinal microbiota to promote the progression of PD is not well understood. Due to the diversity and complexity of the mammalian microbiome, understanding and harnessing the potential of the microbiota presents a challenging task. *Drosophila* has a relatively simple commensal community and is feasible for genetical manipulation [23, 24], making it a powerful and experimentally tractable system to study the effect of microbiome on PD pathology. The midgut of adult *Drosophila* is maintained by intestinal stem cells (ISCs) and their undifferentiated daughters (referred to as enteroblasts, EBs) [25]. ISCs can self-renew and directly differentiate into two major epithelial cell types: absorptive enterocytes and secretory enteroendocrine cells. In this study, we tested the proliferation and differentiation of ISCs, and investigated gastrointestinal deficits and physiological changes under the condition of human α-syn overexpression in *Drosophila* midgut.

## Materials and methods

### Fly husbandry and stocks

All fly stocks were raised at 25 °C and 60% humidity in a 12/12 h light/dark cycle on standard cornmeal-yeast-sucrose food unless otherwise noted. Antibiotic food was prepared by adding 150 μg/ml carbenicillin, 150 μg/ml metronidazole, and 75 μg/ml tetracyclin. The line of *esg-Gal4* was combined with a ubiquitously expressed temperature-sensitive Gal80 inhibitor (*esg-Gal4*; *tub-Gal80*^*TS*^) as described [20, 26]. Crosses and flies were kept at 20–21 °C, then shifted to 29 °C for 3–7 days to allow expression of the transgenes in ISCs and EBs. *UAS-Bsk*, *UAS-Jun*^*bZIP*^ [27], *UAS-GFP*, *UAS-Synuclein*^*A30P*^ and *TH-GAL4* were purchased from the Bloomington *Drosophila* Stock Center. The *ddc-gal4, UAS-Synuclein* (III) line was gifted from Professor Tong Wey Koh in Temasek Life Sciences Laboratory, Singapore. The detailed information of fly stocks is available in Additional file 1: Table S1.

### Drug treatment

Rotenone (Sigma-Aldrich, St. Louis, MO) was initially solubilized to 50 mM in DMSO, and diluted to 7.5 mM in warm liquid fly food [28]. Adult flies were raised on vehicle/rotenone food from 7 days old. The antioxidant, N-acetylcysteine amide (Sigma-Aldrich), was dissolved in rotenone-containing fly food at a concentration of 20 mg/ml.

### A dye penetration test (smurf)

The barrier defect assay (smurf assay) was conducted as described previously [29, 30]. Briefly, more than 80 flies were maintained on standard food until the day of the smurf assay. Twenty flies per test vial were transferred to a standard food with Blue dye no. 1 (Sigma-Aldrich, 2.5% *m*/*v*). Flies were reared on the medium with dyes for 12 h. The proportion of animals with a phenotype indicative of epithelial barrier dysfunction was subsequently monitored.

### Climbing assay

The rapid iterative negative geotaxis assay was carried out according to previous protocols [31, 32]. To analyze climbing ability, 15 flies were transferred into each cylinder (inner diameter: 22 mm; height: 170 mm) by gentle aspiration. An initial mechanical shock was applied to tap all of the flies down to the bottom of the cylinder. Each climb was recorded for 2 min using a video camera. Climbing distances were calculated using the RflyDetection software.

### Lifespan and survival assay

All experiments were repeated at least three times, and the proportion of surviving flies was calculated at each time point of the experiment. Newly eclosed male and female flies (1–2 days old) were separately collected and placed into vials (20 flies/vial), and each group contained 3 to 5 vials, resulting in 60–100 flies in each group. The flies were moved to fresh vials with standard fly food every 3 or 4 days, and the number of dead flies was counted. Survival curves were generated using the Prism software.

### Real-time PCR analysis

Fly intestines were dissected and transferred to cold phosphate-buffered saline (PBS) buffer. A total of 40 intestines were homogenized with TRIzol reagent (Thermo Fisher, Waltham, MA), and RNA was extracted as previously described [29]. Ten micrograms of total RNA were reversely transcribed using SuperScript IV First-Strand Synthesis System Kit (Thermo Fisher). The primer sequences are shown in Additional file 2: Table S2. Gene expressions were assessed using a Light Cycler 96 (Roche, Basel, Switzerland). The ΔCt method was used to analyze data, using *rp49* as the reference gene. The relative expression values were calculated using the following formula: ΔCt = Ct (target gene)—Ct (reference gene), and the relative expression was equal to 2^−ΔΔCt^.

### Immunofluorescence staining

The immunofluorescence assay was performed as described [33]. Flies were fed with 5% sucrose solution with or without rotenone for 12 h. Intestines were dissected in PBS and fixed with 4% paraformaldehyde for 25 min at room temperature. The samples were blocked with a blocking solution (PBS with 0.3% Triton X-100, 0.2% goat serum and 0.1% fetal calf serum) for 25 min, incubated in primary antibodies overnight at 4 °C, and washed in PBS supplemented with 0.3% Triton X-100 (PBST). Then the samples were incubated with the secondary antibodies conjugated with Alexa 488 or 568 (Thermo Fisher, 1:1000, Waltham, MA) for 2 h at room temperature. Primary antibodies were α-syn (Novus Biologicals, 1:1,000, Centennial, CO), phospho-histone 3 (Millipore, 1:1,000, Burlington, MA), Dlg (DHSB, 1:50, Iowa City, IA) and phospho-Jun N-terminal Kinase (phospho-JNK) (Millipore, 1:200), while nuclear DNA was detected by DAPI (Thermo Fisher). All immunostained samples were mounted with Vectashield (Vector Laboratories, Newark, CA), and images were observed by confocal microscopy (Olympus Fluoview FV3000, Shinjuku, Tokyo, Japan) and processed using Adobe Photoshop (San Jose, CA).

### Bacterial culture and counting of colony-forming units

The preparation of mixed bacteria from conventionally reared adult flies was performed as described [23, 24]. The fly-associated strain of *Lactobacillus plantarum* with Genbank accession number KY038178 was used. Mixed bacteria and *L. plantarum* were cultured in Luria–Bertani (LB) and De Man, Rogosa and Sharpe (MRS) broth at 30 °C, respectively. Bacterial cells were harvested by centrifugation, washed twice with PBS, and resuspended in PBS to the concentration of 10^8^ cells/ml (OD_595_ = 1). Bacterial suspensions (0.1 OD or ~ 10^7^ cells) were placed into autoclaved fly food with germ-free (GF) embryos to generate gnotobiotic flies. To quantify the number of bacteria associated with the flies, 15 adult flies were surface sterilized using 70% ethanol for 1 min, and then homogenized with a pestle in a 1.5-ml tube with 200 μl PBS. The bacterial loads were calculated by plating fly homogenates on LB or MRS agar plates. The plates were then incubated at 30 °C for 48 h, and the numbers of colony-forming units were counted at 48 h after incubation.

### Western blotting

Twenty guts from adult flies were dissected in ice-cold PBS, and homogenized in 50-μl lysis buffer followed by mixing with  5× loading buffer as described previously [33]. Ten microliters of total lysates were subjected to Western blotting. The primary antibodies were anti-α-syn (Novus Biologicals, 1:2000 ) and anti-α-actin (Millipore, 1:50,000). The processed membrane was developed with chemiluminescent HRP substrate (Millipore, 1:1000) to detect target proteins.

### 16S DNA sequencing

High-throughput sequencing of 16S DNA was carried out according to a previously described protocol [24]. Briefly, 30 adult female fly midguts were dissected into ice-cold PBS, and frozen samples of intestines were sent to the Novogene Bioinformatics Technology Co., Ltd. (Beijing, China). Total bacterial DNA extraction and sequencing were conducted according to standard protocols. DNA was amplified using the 515f/806r primer set (515f : 5′-GTG CCA GCM GCC GCG GTA A-3′, 806r: 5′-XXX XXX GGA CTA CHV GGG TWT CTA AT-3′), which targets the V4 region of the bacterial 16S rDNA. Pyrosequencing was conducted on an Illumina MiSeq 2 × 250 platform (San Diego, CA) according to published protocols. Sample reads were assembled using mothur v1.32. Chimeric sequences were removed using the USEARCH software based on the UCHIME algorithm. The microbial diversity was analyzed using the QIIME software with Python scripts. Operational Taxonomic Units (OTUs) were picked using the de novo OTU picking protocol, with a 97% similarity threshold.

### RNA sequencing

Briefly, 40 female middle intestines were dissected into ice-cold diethyl pyrocarbonate (DEPC)-treated PBS, and sent to the Novogene Bioinformatics Technology Co., Ltd. (Beijing, China). Total RNA (~ 1 μg) for each sample was used for cDNA synthesis. cDNA libraries were constructed using NEB Next Ultra II DNA library prep kits (New England Biolabs, Ipswich, MA) and adapters in VAHTS DNA Adapters set1/2 for Illumina (Vazyme, Nanjing, China). Sequencing was carried out on an Illumina Hiseq-2500 sequencing system with a 50-bp read length, according to the manufacturer’s instructions. The single-end RNA-seq raw reads were aligned against the *D. melanogaster* genome (BDGP6) using STAR (v020201). The total number of reads was assigned to protein-coding genes using feature Counts (v1.6.3). The GOseqR package, based on the Wallenius non-central hyper-geometric distribution, was used to determine Gene Ontology (GO) enrichment of the differentially expressed genes (DEGs). Kyoto Encyclopedia of Genes and Genomes (KEGG) is a database resource for understanding high-level functions (http://www.genome.jp/kegg/). The KOBAS software was implemented to assess the statistical enrichment of differentially expressed genes in KEGG pathways.

### In vivo detection of reactive oxygen species (ROS)

ROS generation was examined using the intracellular ROS-sensitive fluorescent dye dihydroethidium (Thermo Fisher) [29]. The midguts of young or aged flies were dissected in PBS and incubated in 5-μM dihydroethidium for 30 min at room temperature in the dark. The midguts were then washed three times with PBS, and were immediately fixed with 4% paraformaldehyde for 10 min at room temperature. The samples were washed three times with PBS. Subsequently, the samples were transferred to a glass slide with a drop of PBS for microscope observation (Olympus Fluoview FV3000). The intensity of immunofluorescence was quantified using ImageJ software (V1.53, Bethesda, MD).

### H_2_O_2_ assay

Fly intestines were dissected in ice-cold PBS, and 15 midguts were homogenized in 200-μl lysis buffer. After homogenization, the samples were centrifuged at 12,000*g*  at 4 °C for 5 min, and the supernatant was collected for H_2_O_2_ assay. The production of H_2_O_2_ was examined using the Peroxide Assay Kit (Sigma, MAK311-1KT) according to the manufacturer’s instructions. The concentration of H_2_O_2_ released was calculated according to a standard curve of hydrogen peroxide. The measurement was repeated three times.

### Statistical analysis

All analyses were performed in the GraphPad Prism statistical software (v9.1.). Specific statistical tests are noted for individual experiments in the Results section. Survival curves were compared with log-rank tests, with Bonferroni corrections for *P* values where multiple comparisons were necessary. All survival and lifespan graphs represent one out of three independent repeats with 4–5 cohorts of 20 flies per genotype. The *P*-values of the other experimental groups were calculated using one-way ANOVA with Bonferroni multiple-comparison test. Data are presented as mean ± standard error of the mean (S.E.M.). *P < 0.05, **P < 0.01, and ***P < 0.001.

## Results

### Transgenic expression of α-syn in *Drosophila* midguts

**Fig. 1:**
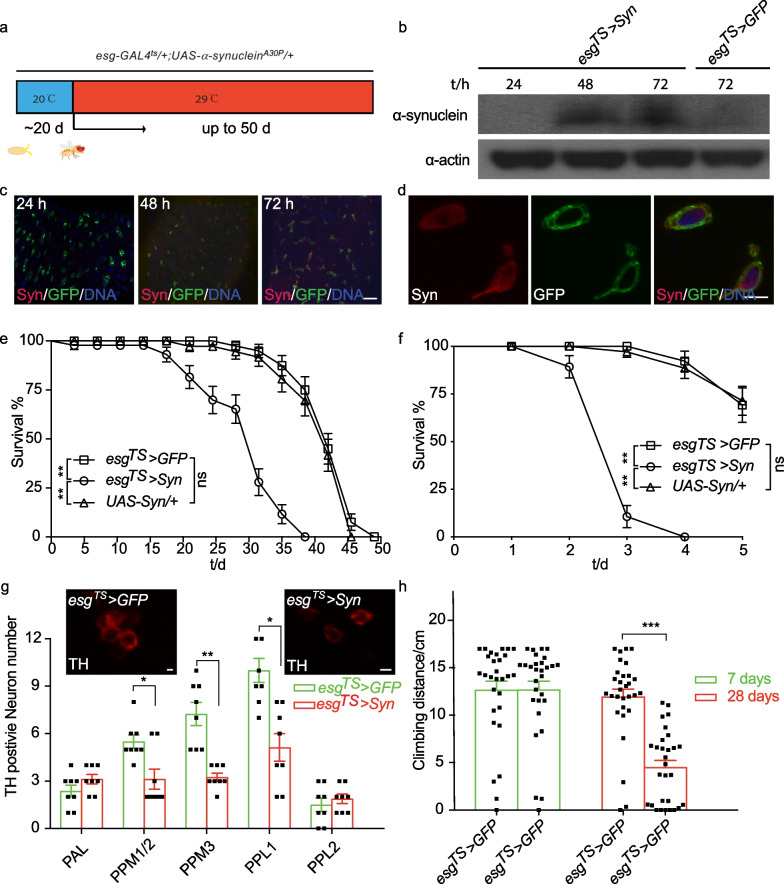
Intestinal overexpression of α-syn recapitulates Parkinson’s disease pathology in *Drosophila.* **a** Diagram of the temporal expression of the α-syn transgene in the *Drosophila* intestine. Temperature-controlled expression of *UAS-Syn* or *UAS-GFP* was achieved by co-expression of *esg-GAL4* with a temperature-sensitive version of the GAL4 inhibitor *Gal80*^*TS*^, in which the expression of UAS-synuclein was inactivated and induced at 18 °C and 29 °C, respectively. We refer to the two genotypes as *esg*^*TS*^ > *Syn* (*w; esg-Gal4, UAS-GFP/*+*; UAS-Syn/tubulin-Gal80*^*TS*^)  and *esg*^*TS*^ > *GFP* (*w; esg-Gal4, UAS-GFP/*+*; UAS-GFP/tubulin-Gal80*^*TS*^)*.* **b** Western blot showing the expression of human α-syn in the midgut of adult flies after 48 h at 29 °C. α-Actin was used as a loading control. **c** Immunostaining showing the expression of human α-syn in fly intestinal stem cells (Scale bar, 1 mm). α-Syn expression in intestinal stem cells increased following a shift to 29 °C. **d** Representative images showing exogenous α-syn in both cytoplasm and nucleus (Scale bar, 1.5 μm). Red: α-syn; green: GFP; blue: DNA. **e** Decreased lifespan of intestinal PD flies. Adult flies were cultured as described in panel **a** and the percentage of living male flies was calculated to monitor lifespan. **f** Survival rates of intestinal PD flies challenged with rotenone. Sucrose solution containing 7.5 mM rotenone was used for survival assay. **g** Intestinal overexpression of α-syn causes an age-dependent dopaminergic (DA) neuron loss. Numbers of TH-positive DA neurons in five clusters of each genotype were counted and analyzed (bottom). Representative images of TH-positive DA neurons in the PPM1 cluster of 28-day-old control or intestinal α-syn-expressing flies (top). Scale bars, 0.2 μm. **h** Expression of α-syn in the midgut leads to progressive locomotor deficits. For the locomotion assay, climbing distances of each genotype of males at different time points are plotted as bar graphs. Mean ± SEM; *P*-values of survival curves were calculated using log-rank tests (using total fly numbers), and for category graphs using one-way ANOVA with a Bonferroni multiple-comparison test. *P < 0.05; **P < 0.01; ***P < 0.001

There is no homologue of α-syn in *Drosophila*, which makes *Drosophila* a feasible model to investigate the role of human α-syn in PD pathologies. Feany and Bender (2000) developed the first transgenic model of PD in *Drosophila* through ectopic expression of wild-type or pathogenic mutant forms of human α-syn in neurons [34]. However, the potential role of intestinal α-syn in PD pathogenesis in *Drosophila* is largely unknown. To address this question, we acutely overexpressed human α-syn in adult *Drosophila* midguts by manipulating *esg-GAL4* and the temperature-sensitive GAL4 repressor, *tub-Gal80*^*TS*^ (Fig. 1a). This *esg-GAL4* line simultaneously allows temporal expression of *UAS-GFP* that links with ISCs and EBs of flies (*w; esg-Gal4, UAS-GFP/* + *; UAS-Synuclein/tubulin-Gal80*^*TS*^) without other developmental defects [35]. Expression efficiencies of these transgenes were validated by western blot using an antibody against human α-syn. As shown in Fig. 1b, we found that the 48-h shift to a restrictive temperature (29 °C) triggered an accumulation of α-syn in the intestines of *esg-GAL4* > *UAS-*α-*syn* flies (hereafter termed *esg*^*TS*^ > *Syn*), but not in control flies expressing green fluorescent protein (GFP) (termed hereafter *esg*^*TS*^*> Syn*). To visualize the expression pattern of α-syn in *Drosophila* intestines, we performed immunofluorescence staining against α-syn over time, and observed robust expression of α-syn in esg-positive cells after 48 h at 29 °C (Fig. 1c). Zoomed images showed that α-syn was primarily cytosolic, but also in the nucleus (Fig. 1d), reminiscent of the localization of α-syn in human cells [36]. Consistent with the previous study [37], the *esg-GAL4* driver did not drive detectable expression of GFP in the brain after adult eclosion (Additional file 5: Fig. S1a), suggesting that the brain-derived α-syn is unlikely to directly influence PD pathogenesis in the brain. Therefore, the *Drosophila* model with intestinal expression of human α-syn enables us to directly examine the potential role of α-syn in intestinal homeostasis and progression of PD.

### Intestinal expression of α-syn recapitulates the disease phenotypes of PD

Previous studies, including our work, have demonstrated that PD flies expressing α-syn or other proteins in neurons exhibit impaired lifespan, accelerated age-dependent motor defects and loss of DA neurons [28, 38]. First, we assessed whether the intestinal expression of α-syn affects *Drosophila* lifespan, because PD brings an increased risk of earlier death. The results showed that the median survival time of *esg*^*TS*^ > *Syn* flies was much shorter than that of control flies (Fig. 1e). To rule out the possibility that *UAS-Syn* has off-target effects, we also examined the lifespan of *UAS-Syn/* + flies as another control. The result showed that the lifespan of *UAS-Syn/* + flies was significantly longer than that of the *esg*^*TS*^ > *Syn* flies, and similar to that of *esg*^*TS*^ > *GFP* (Fig. 1e), suggesting that this impaired lifespan did not arise from genetic variation. To save time and labor, we mainly used *esg*^*TS*^ > *GFP* as the control in later experiments. The multiple lines of evidence showing that environmental toxin induces PD-like symptoms prompted us to analyze the survival of flies under rotenone challenge. Consistently, the *esg*^*TS*^ > Syn flies manifested a shortened lifespan in the presence of rotenone (Fig. 1f), indicating that the intestinal α-syn reduces the life expectancy of *Drosophila* challenged with stress. A hallmark of PD pathology is the progressive loss of DA neurons, which is associated with behavioral dysfunction in PD patients. *Drosophila* has six paired DA neuron clusters located in the posterior protocerebrum and the anterior protocerebrum [39]. We further found that the 28-day-old flies with intestinal expression of α-syn had significantly lower numbers of tyrosine hydroxylase-positive (TH^+^) cells in the protocerebral posterior median (PPM) clusters 1/2 and 3, and the protocerebral posterior lateral (PPL) 1 cluster, compared with age-matched control flies, but not in other clusters (Fig. 1g). Notably, 7-day old *esg*^*TS*^ > *Syn* flies showed no difference in the numbers of TH^+^ cells compared to control flies. Consistently, there was a decrease in the number of GFP-positive cells driven by *TH-GAL4* in the PPM clusters 1/2 and 3, and the PPL1 cluster of aged *esg*^*TS*^ > *Syn* flies compared to control (Additional file 5: Fig. S1b). These results suggest that intestinal expression of α-syn accelerates the age-dependent DA neuron loss in *Drosophila* brains. Finally, we assessed the effect of intestinal α-syn on *Drosophila* locomotion with adult climbing assays. Negative geotaxis is used to examine how quickly a fly can climb vertically after being tapped to the bottom of a vessel. At the age of 7 days, little difference in climbing distance was observed between flies expressing α-syn or GFP (Fig. 1h). However, the 28-day-old *esg*^*TS*^ > *Syn* flies climbed more slowly than control flies. This result indicated that the intestinal expression of α-syn results in an accelerated age-dependent decline in climbing ability, reminiscent of the age-dependent locomotor deficits in PD patients. Taken together, the above results demonstrated that the intestine-derived α-syn recapitulates the disease phenotypes of PD in *Drosophila*.

### Transgenic expression of α-syn causes intestinal dysplasia

**Fig. 2:**
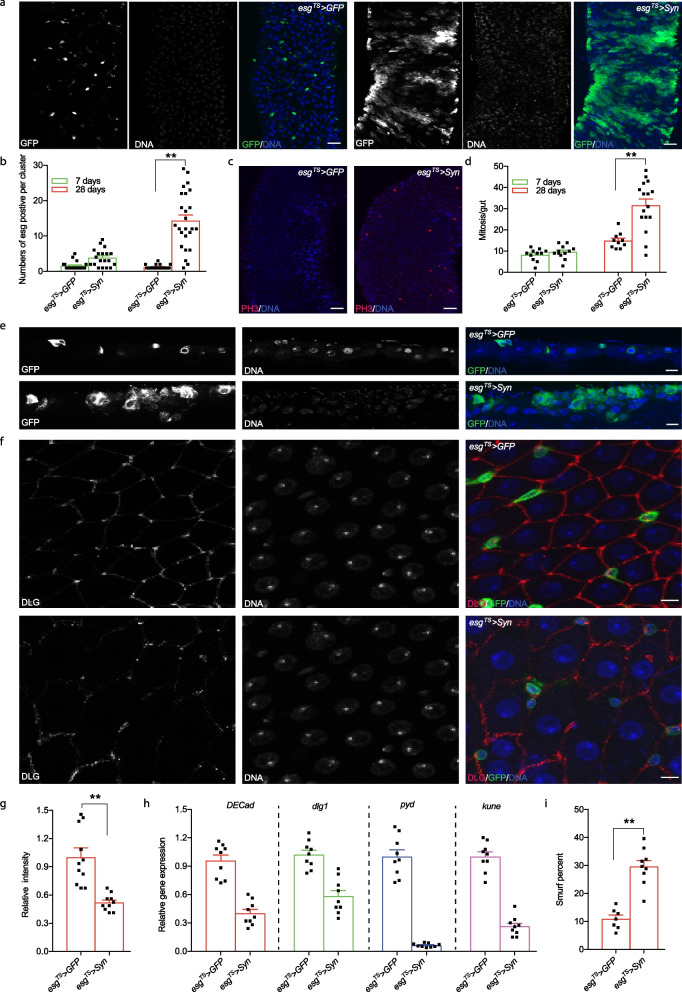
Intestinal overexpression of α-syn causes dysplasia in the *Drosophila* intestine. **a** Representative images of the anterior midguts of *esg*^*TS*^ > *GFP* and *esg*^*TS*^ > *Syn* flies after 28 days at 29 °C. Intestinal stem cells (ISCs) and enteroblasts (EBs) were labelled by GFP expression (green), and DNA was detected using DAPI staining (blue). **b** Quantification of cells in esg-positive cell clusters in the anterior midgut. Scale bars: 100 μm. **c**, **d** Increased cell proliferation per adult midgut in flies expressing α-syn. Representative images of gut expressing α-syn and GFP. Red, Phospho-histone H3; blue: DNA; Scale bars, 100 μm. **e** The disorganized architecture of the intestinal epithelium. Cross-sections of intestines illustrate the loss of the monolayered structure of the intestinal epithelium in *esg*^*TS*^ > *Syn* flies. ISCs and EBs were GFP-positive (green), and DNA was detected using DAPI (blue). Scale bars, 1 μm. **f**, **g** Representative images (**f**) and quantification (**g**) of anti-Dlg staining of the posterior midgut. The intercellular junction was unpaired. Green, GFP; Red, anti-Dlg; blue, DNA; scale bars, 1 μm. **h** Junction protein gene expression assayed by qPCR from middle intestines of *esg*^*TS*^ > *Syn* and *esg*^*TS*^ > *GFP* flies. *DECad*, *Drosophila E-Cadherin*; *dlg1*, *disc large 1*; *pyd*, p*olychaetoid*; *kune*, *kune*-*kune*. **i** Intestinal overexpression of α-syn impairs the integrity of the *Drosophila* intestine. Smurf assay for intestine barrier defects was assessed in *esg*^*TS*^ > *Syn* and *esg*^*TS*^ > *GFP* flies on the blue dye-containing food. *n* is denoted as the number of spots on each column on the plot. Mean ± SEM; the *P*-values were calculated using one-way ANOVA with a Bonferroni multiple-comparison test. *P < 0.05; **P < 0.01; ***P < 0.001

Given that soluble α-syn has a propensity to form toxic oligomers to disrupt membrane integrity and function [40], we further examined the effect of transgenic α-syn on intestinal homeostasis. Strikingly, we observed a significant increase in the number of esg-positive cells (GFP-positive cells) in aged *esg*^*TS*^ > *Syn* flies (after 28 days at 29 °C) compared with control flies, indicative of prominent proliferation and/or differentiation defects in the ISC lineage (Fig. 2a, b). Moreover, more mitotic cells by using phospho-histone3 (PH3) antibody were observed in aged *esg*^*TS*^ > *Syn* intestines compared to the counterparts (Fig. 2c, d), consistent with the increased mitosis after α-syn inoculation observed in mice [13]. Interestingly, we observed that the size of the esg-positive cells varied significantly, approaching the size of enterocytes in some cases (Fig. 2e), suggesting that human α-syn resulted in differentiation defects in the *Drosophila* ISC lineage. The fly intestine consists of a simple epithelium surrounded by visceral muscles, nerves, and the trachea. Rather than forming an ordinary monolayered epithelium, the intestinal cells of *esg*^*TS*^ > *Syn* clustered and overlapped in the apicobasal axis, and formed multilayered tissues (Fig. 2e), suggesting that apicobasal organization of the epithelium was disrupted by intestinal α-syn. We further examined intestinal junctions using the septate junction protein Disc large (Dlg). Indeed, Dlg was either mislocalized or reduced in *esg*^*TS*^ > *Syn* flies (Fig. 2f, g), suggesting that the septate junctions  were impaired in their guts. We confirmed this result by assessing the expression levels of *Drosophila* genes associated with the junctions of the intestine [41]. Indeed, *DECad*, *dlg1*, *pyd*, and *kune* in *esg*^*TS*^ > *Syn* flies showed a significant decrease in their transcriptional expression (Fig. 2h). The overall altered morphologies of the intestinal epithelium correlate with a general functional deterioration of the intestinal mucosa, prompting us to explore whether α-syn affects the integrity of intestines by using the “smurf” assay as previously described [29, 30]. In the presence of unabsorbable blue dye, loss of intestinal barrier function (referred to as intestinal leakage) allows the unabsorbable dye to go across the gut barrier. Indeed, our data showed that the *esg*^*TS*^ > *Syn* flies had a significantly higher fraction of blue dye leakage compared to their controls (Fig. 2i). Taken together, these results suggest that α-syn contributes to the age-associated onset of intestinal barrier structures and dysfunction.

### Intestinal α-syn alters immunity and metabolic profile

**Fig. 3:**
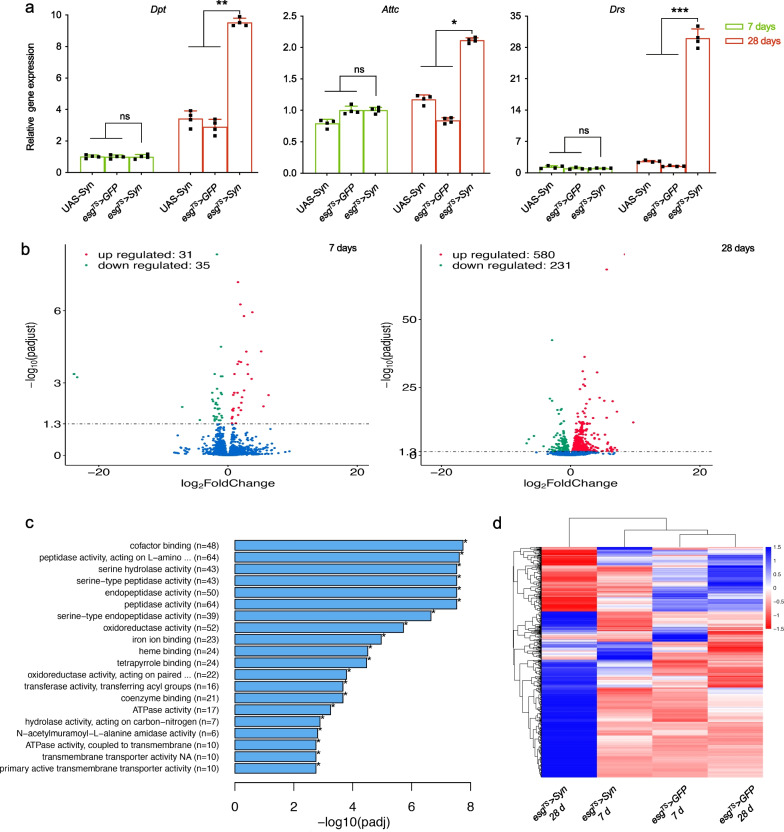
Intestinal overexpression of α-syn causes alterations of transcription profiles and microbiota composition. **a** Activation of intestinal immunity by intestinal α-syn. Antimicrobial peptide gene expression in the middle intestine of 28-day-old *Drosophila* using qPCR. *Dpt*, *Diptericin*; *Attc*, *Attacin;* *Drs*, *Drosocin*. *n* = 4. **b** Volcano plot comparing gene expression profiles of *esg*^*TS*^ > *Syn* and *esg*^*TS*^ > *GFP* in fly intestines. Red dots depict genes highly upregulated in *esg*^*TS*^ > *Syn* intestines (log2 fold change < 1; adjusted *P* < 0.01), and green dots depict genes significantly downregulated in *esg*^*TS*^ > *Syn* intestines (log2 fold change < 1; adjusted *P* < 0.01). Blue dots depict genes without significant alteration. **c** Gene ontology (GO) analysis of the significantly altered genes in *esg*^*TS*^ > *Syn* intestines. The top 20 GO terms for the 2231 upregulated genes are shown in red. The heights of the columns represent the alteration level of gene expression in this GO term. **d** Supervised hierarchical clustering of genes based on RNA-Seq scores. The relative expression levels of clustered genes are shown on the right on a scale of -1.5 (downregulated) to 1.5 (upregulated)

Given that dysplasia triggers immune activation [26], we set out to assess changes in the expression of antimicrobial peptides in the intestines. Using quantitative PCR (qPCR), we observed an increase in the expression of *dpt*, *Attc*, and *Drs* in aged *esg*^*TS*^ > *Syn* intestine compared to their controls (Fig. 3a). To investigate the global changes in gene expression caused by intestinal α-syn, we conducted gene expression analysis of middle intestines using RNA sequencing (RNA-seq). Compared to wild-type control, the *esg*^*TS*^ > *Syn* intestines displayed significant upregulation of 580 genes and significant downregulation of 231 genes (Fig. 3b, Additional file 3: Table S3). GO analysis revealed that the majority of the top 20 altered gene categories were related to amino acid biosynthesis, oxidative stress, mitosis, and energy metabolism (Fig. 3c), which was consistent with the accelerated ageing of intestines. Unsupervised hierarchal clustering analyses revealed that the expression profile of highly viable genes in *esg*^*TS*^ > *Syn* intestines formed a distinct cluster compared with wild-type controls and the two other corresponding young groups (Fig. 3d). Our results are consistent with a recent study that shows an altered profile of proteomics in heads from α-synucleinopathy model flies [42]. Taken together, our data suggest that the intestinal expression of α-syn widely alters expression of genes associated with innate immunity and metabolism.

### Intestinal α-syn causes dysbiosis

**Fig. 4:**
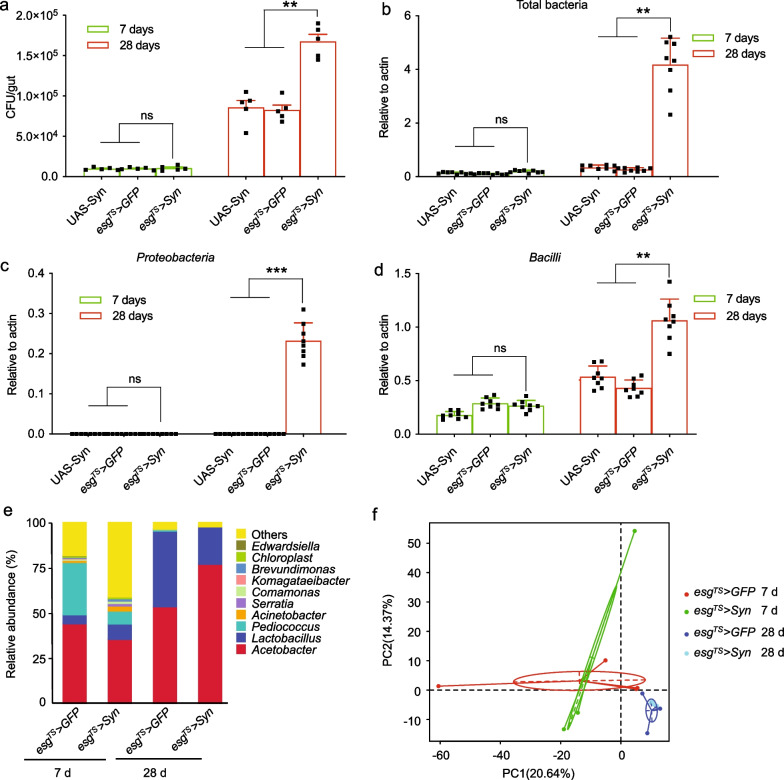
Intestinal overexpression of α-syn causes dysbiosis. **a** The total bacterial load in *Drosophila* intestines. Midgut cultivable bacterial load in 7- and 28-day-old adult flies. Bacterial load was determined by plating the homogenate of midguts diluted 1000 × onto LB agar plates. **b**–**d** The relative abundance of total bacteria (**b**), *Proteobacteria* (**c**) and *Bacilli* (**d**). Bacterial levels were assayed using qPCR of the 16S rRNA gene in the middle intestine 7 or 28 days after eclosion. **e** Proportional sequence assignments for the taxonomic units of intestinal bacteria. The legend shows color-coded assignments and taxonomic hierarchy. **f** Principal coordinate analysis (PCA) of unweighted, jack-knifed UniFrac distances of bacterial communities in fly intestines. PC1, principal coordinate 1; PC2, principal coordinate 2. Mean ± SEM; *P*-values were calculated using one-way ANOVA with Bonferroni multiple-comparison test. *P < 0.05; **P < 0.01; ***P < 0.001

Since dysbiosis is closely associated with ageing and mortality, we propose that ectopic expression of α-syn affects the homeostasis of microbiota in *Drosophila* intestines. The cultivable bacterial loads in *Drosophila* midguts were assessed by plating fly homogenates on nutrition agar plates. The total bacterial load was dramatically elevated in 28-day-old *esg*^*TS*^> *Syn* flies compared with controls (Fig. 4a), while the loads were comparable among young groups. The result of qPCR further showed that the total bacterial load in aged *esg*^*TS*^> *Syn* flies was significantly higher (∼3.7-fold) compared with controls (Fig. 4b), while the loads were comparable among young groups. More importantly, *Proteobacteria* showed a striking increase (~6300-fold) in *esg*^*TS*^> *Syn* flies (Fig. 4c), while they were comparable in young groups. However, *Bacilli* showed a moderate increase (~2.7-fold) even in aged *esg*^*TS*^> *Syn* flies (Fig. 4d). To further investigate global changes in the intestinal microbial composition, we conducted 16S rDNA sequencing on *esg*^*TS*^> *Syn* and control intestines. In young flies, the midgut bacteria were diverse and dominated by at least five genera: *Acetobacter*, *Pediococcus*, *Lactobacillus*, *Actinetobacter*, and *Serratia* (Fig. 4e and Additional file 4: Table S4). However, the diversity of microbiota was remarkedly lower in the aged flies, with the dominance of *Acetobacter* and *Lactobacillus*. The proportional representation of each taxon clearly illustrated the increase of *Acetobacter* (78.3%) in the *esg*^*TS*^> *Syn* intestines, while both *Acetobacter* (52.5%) and *Lactobacillus* (40.6%) predominated in the control intestines (Fig. 4e). This result suggested that the composition and diversity of the midgut bacterial population are markedly altered *in esg*^*TS*^> *Syn* flies. Moreover, principal component analysis showed that the intestinal microbiome cluster in aged *esg*^*TS*^> *Syn* flies dramatically differed from those of the age-matched control flies (Fig. 4f). Taken together, our findings suggest that intestinal α-syn results in alterations of the component of gut microbiota.

### Removal of microbiota delays the progression of PD

**Fig. 5:**
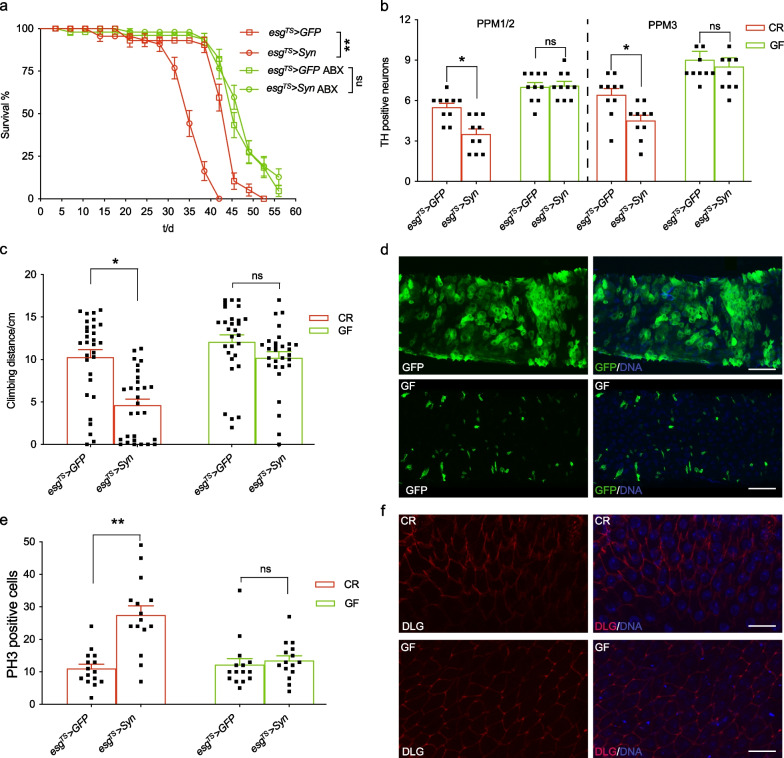
Dysbiosis aggravates the pathology of Parkinson’s disease in the intestine. **a** Bacterial depletion attenuated the impairment of lifespan of intestinal *Syn* flies. Germ-free (GF) flies were generated with fly diet with antibiotics cocktail (carbenicillin, metronidazole, and tetracyclin). Adult flies were cultured as described in Fig. 1a, and lifespan curves were recorded. **b** Bacterial depletion alleviated the age-dependent DA neuron loss induced by intestinal α-syn. **c** Antibiotics improved the progressive locomotor deficits induced by intestinal α-syn. **d** Antibiotics inhibited the intestinal α-syn-induced dysplasia of midgut. Green, GFP; blue, DNA; Scale bars, 2 μm. **e** Bacterial depletion decreased the number of phospho-H3-positive cells in midgut. **f** Bacterial depletion attenuated the interruption of intercellular junction. Red, anti-Dlg; blue, DNA; Scale bars, 0.5 μm. Mean ± SEM; *P*-values for survival curves were calculated using log-rank tests (using total fly numbers), and for category graphs using one-way ANOVA with Bonferroni multiple-comparison test. *P < 0.05; **P < 0.01; ***P < 0.001. CR: conventionally reared; PH3: phospho-H3

Next, we set out to determine whether preventing the age-related onset of dysbiosis in adult flies may decelerate the progression of PD. To this end, flies were fed with a diet containing mixed antibiotics from day 15 of adulthood to generate GF flies (Additional file 5: Fig. S2a). Indeed, *esg*^*TS*^ > *Syn* flies treated with antibiotics showed a comparable lifespan to wild-type controls (Fig. 5a), indicating that the microbiota aggravate α-syn-mediated mortality in *Drosophila*. Moreover, this antibiotic treatment alleviated the loss of DA neurons in the PPM clusters 1/2 and 3 as well as the PPL1 cluster in *esg*^*TS*^ > *Syn* flies (Fig. 5b). The age-related locomotor decline of *esg*^*TS*^ > *Syn* flies was consistently ameliorated in the presence of antibiotics (Fig. 5c). In addition, we found that the antibiotic treatment decreased the numbers of esg-positive cells in each cluster (Fig. 5d and Additional file 5: Fig. 2b) and PH3-positive cells (Fig. 5e) in aged *esg*^*TS*^ > *Syn* flies, suggesting that the α-syn-mediated proliferation depends on dysbiosis. Mislocalization of Dlg in *esg*^*TS*^ > *Syn* intestines was also effectively prevented by antibiotic treatment (Fig. 5f and Additional file 5: Fig. S2c). In addition, the levels of *Dpt*, *Attc* and *Drs* were lower in mixed antibiotics (ABX)-treated flies compared to control groups (Additional file 5: Fig. S2d). Altogether, the results demonstrate that preventing dysbiosis decelerates the progression of intestinal α-syn-induced PD.

### Intestinal α-syn triggers the dual oxidase (DUOX)–ROS–JNK pathway

**Fig. 6:**
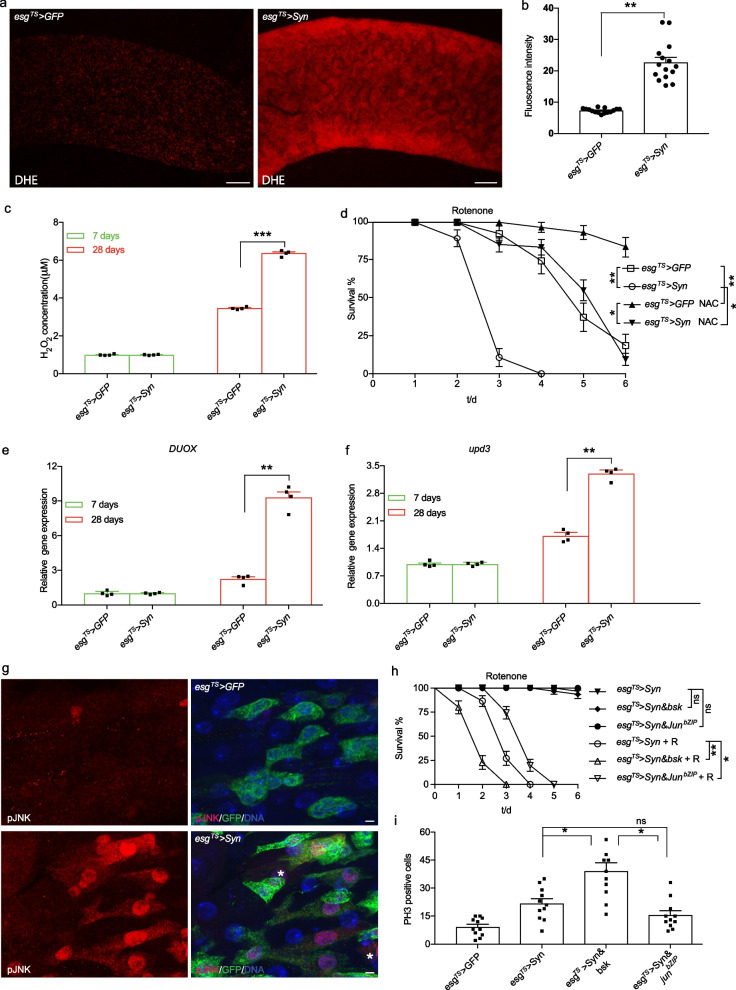
Intestinal α-syn triggers the DUOX–ROS–JNK pathway. **a**, **b** Levels of reactive oxygen species (ROS). Fluorescence staining for peroxidase activity using dihydroethidium (DHE) in fly midgut (**a**). The quantification of fluorescence intensity (**b**). Scale bars, 100 μm. **c** The level of H_2_O_2_ was elevated by intestinal α-syn. ROS activity was detected by the H_2_O_2_ assay. **d** Survival rates of intestinal PD flies challenged with rotenone, with or without ROS-scavenging N-acetylcysteine treatment. **e**, **f** Activation of DUOX and JNK signal by the overexpression of α-syn in guts. The levels of *Duox* and *upd3* of the middle intestine were assessed in 28-day-old flies with RT-PCR. **g** An increase of phospho-JNK level in *esg*^*TS*^ > *Syn* intestine. Green, GFP; Red, anti-phospo-JNK; blue, DNA; asterisk, esg-negative and p-JNK-positive cells; scale bars, 0.2 μm. **h** JNK signaling is required for the attenuation of lifespan of *esg*^*TS*^ > *Syn* flies treated with rotenone. A dominant-negative form of Jun (*Jun*^*bZIP*^) and a wild-type form of Basket (*bsk*) were used to inhibit and activate the JNK signaling in midguts, respectively. R: rotenone. **i** JNK-mediated proliferation of intestinal stem cells in *esg*^*TS*^ > *Syn* intestines. Mean ± SEM; the *P*-values for survival curves were calculated using log-rank tests (using total fly numbers), and for category graphs using one-way ANOVA with Bonferroni multiple-comparison test. *P < 0.05; **P < 0.01; ***P < 0.001; ns, not significant

Given that DUOX is responsible for the dysbiosis-induced generation of ROS [43], it is conceivable that high microbial burdens aggravate the progression of PD through the DUOX–ROS pathway. To address it, we examined the levels of ROS in the midgut epithelium. Indeed, ROS activity measured by dihydroethidium staining was higher in *esg*^*TS*^ > *Syn* flies than that of control flies (Fig. 6a, b). Consistent with this finding, the level of H_2_O_2_ in aged *esg*^*TS*^ > *Syn* flies was relatively higher than that of control flies (Fig. 6c). Additionally, ROS scavenger N-acetylcysteine attenuated the rotenone-induced mortality of *esg*^*TS*^ > *Syn* flies (Fig. 6d). As expected, our results showed that the expression of DUOX was significantly higher in the midguts of *esg*^*TS*^ > *Syn* flies than in control flies (Fig. 6e), suggesting that intestinal α-syn triggers the DUOX pathway. Altogether, our findings suggest that intestinal α-syn exacerbates the severity of PD through the DUOX–ROS pathway.

Activation of the JNK pathway in ISCs is required for compensatory cell proliferation in ageing and injury in both insects and mammals [44, 45]. The level of *upd3* consistently increased in the *esg*^*TS*^ > *Syn* intestines compared with control ones (Fig. 6f). To test whether JNK is active in the intestinal epithelium, we checked the levels of phospho-JNK with immunostaining. Indeed, we observed increased phospho-JNK levels in aged *esg*^*TS*^ > *Syn* intestines compared to control intestines (Fig. 6g and Additional file 5: Fig. S3a). Notably, strong levels of JNK activity in *esg*^*TS*^ > *Syn* intestines were detected in esg-positive cells, as well as in enterocytes (~ 10%, Additional file 5: Fig. S3b). Moreover, inhibiting the JNK signaling by a dominant-negative form of *Jun* (*Jun*^*bZIP*^) markedly increased survival of *esg*^*TS*^ > *Syn* flies treated with rotenone compared to *esg*^*TS*^ > *Syn* control flies (Fig. 6h), while activating the JNK signaling by a wild-type form of Basket (*bsk*) decreased the survival of *esg*^*TS*^ > *Syn* flies. Consistently, JNK activation increased the proliferation of intestines in aged *esg*^*TS*^ > *Syn* flies, while JNK suppression decreased it (Fig. 6i). Taken together, we conclude that intestinal α-syn contributes to PD pathogenesis through the DUOX–ROS–JNK pathway.

### Intestinal α-syn aggravates the pathogenesis of brain α-syn-induced PD

**Fig. 7:**
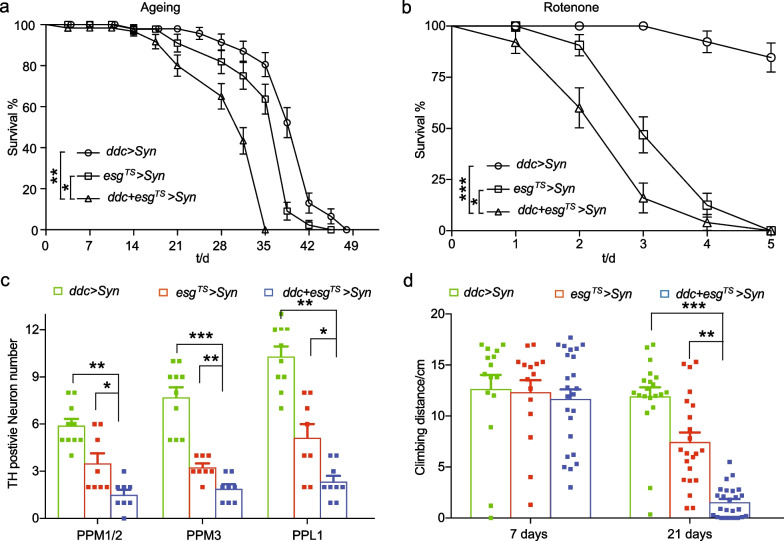
Intestinal α-syn triggers the pathology of Parkinson’s disease mediated by brain α-syn. **a** Exacerbated lifespan of flies with co-expression of α-syn in the brains and the intestine. Intestinal α-syn accelerates the death of flies with brain α-syn. Flies were cultured as described in Fig. 1a and the percentage of living male flies was calculated to monitor lifespan. Flies of *esg*^*TS*^ > *Syn*, *ddc* > *Syn* (*ddc-GAL4, UAS-Syn*) and *ddc* + *esg*^*TS*^ > *Syn* (*esg-GAL4, UAS-GFP/* + ; *ddc-GAL4, UAS-Syn/ tubulin-Gal80*^*TS*^) were used. **b** Survival rates of flies challenged with rotenone. The percentage of living male flies was calculated to monitor survival in the presence of rotenone. **c** Intestinal α-syn exacerbates the age-dependent loss of DA neurons of flies with brain α-syn. The numbers of TH-positive DA neurons in five clusters of each genotype were analyzed in PPM1/2, PPM3, and PPL1 clusters of 28-day-old flies. **d** Intestinal α-syn induces progressive locomotor deficits of flies with brain α-syn. Mean ± SEM; *P*-values for survival curves were calculated using log-rank tests (using total fly numbers), and for category graphs using one-way ANOVA with Bonferroni multiple-comparison test. *P < 0.05; **P < 0.01; ***P < 0.001; ns, not significant

Lines of evidence suggest that α-syn could aggravate the pathology of PD in a vagal nerve-dependent manner [11]. To experimentally examine this concept, human α-syn was overexpressed in both the midguts and dopaminergic neurons using *esg-GAL4* and *ddc-GAL4*. The result showed that the lifespan of flies co-expressing α-syn in intestines and DA neurons was significantly shorter than that of flies with either intestinal expression or DA neuron expression alone (Fig. 7a), indicating that the intestinal expression of α-syn aggravates the pathogenesis of PD in DA neurons. Similarly, the lifespan of flies co-expressing α-syn in guts and DA neurons was shorter than that of flies with either intestinal expression or DA neuron expression alone, when they were challenged with rotenone (Fig. 7b). Moreover, we found that the 21-day-old flies with double expression of α-syn displayed a significant loss of TH^+^ cells compared with age-matched control flies with either intestinal expression or DA neuron expression alone (Fig. 7c), suggesting that α-syn from both guts and brains can synergize to accelerate degeneration of DA neurons in *Drosophila*. At the same time, we observed that flies with dual expression of α-syn exhibited an accelerated decline of locomotor abilities 21 days after post-restriction temperature treatment compared to flies with either intestinal expression or DA neuron expression alone (Fig. 7d). In conclusion, these results suggest that intestinal expression of α-syn aggravates the progression of PD induced by brain α-syn.

## Discussion

The accumulation of toxic forms of α-syn is a key pathological feature of PD [10]. Our current study shows that ectopic α-syn within the intestine is able to produce PD-like pathology in *Drosophila*, including a shorter lifespan, loss of DA neurons, and progressive motor defects (Fig. 1). Consistently, the PPM1/2 cluster that accounts for locomotor deficits and lifespan impairment has a propensity to suffer from greater DA neuron loss upon genetic mutation and environmental toxin [32]. Our findings highlight that intestinal α-syn is capable of triggering the progression of PD in *Drosophila*. Ectopic intestinal α-syn triggers the pathology of PD by disrupting intestinal homeostasis and metabolic profile (Figs. 2, 3). These results corroborate observations of gastrointestinal dysfunction in PD patients, including drooling, gastroparesis, decreased bowel movement frequency, constipation, and anorectal dysfunction [46–48].

Our findings are consistent with observations that gut injection of α-syn fibrils disrupts intestine function and initiates inflammatory responses [13, 14]. More importantly, intestinal α-syn is sufficient to elicit progressive PD-like pathology in *Drosophila*, partially differing from the hypothesis that gut injection of α-syn fibrils has to convert endogenous α-syn in brains to a pathologic species [13, 14]. In the present study, α-syn expression in the gut can independently initiate and aggravate progressive PD-like pathology in the absence of endogenous α-syn in the brain (Fig. 1), and can facilitate progressive PD-like pathology even in the presence of endogenous α-syn (Fig. 7). This enables us to examine the independent role of intestinal α-syn in PD pathogenesis. However, the finding of α-syn propagation from the gastrointestinal tract to the brain in the *Drosophila* model needs to be investigated and validated in mammalian models.

Interestingly, other studies have provided experimental evidence that α-syn aggregates in a prion-like manner [9], supporting the propagation of α-syn from the intestines to the brain in *Drosophila*. In mammals, α-syn is expressed in enteroendocrine cells that directly connect to α-syn–containing nerves [49]. Far from being a passive tube, the gut of adult *Drosophila* provides the first line of defense against pathogens and maintains energy homeostasis by exchanging neuronal and endocrine signals, with other organs [25], partially explaining the potential role of α-syn in PD pathogenesis. The digestive tract of adult *Drosophila* is innervated in three distinct regions, the esophagus-crop-anterior midgut, the midgut-hindgut junction, and the posterior hindgut [50]. The *Drosophila* gut is a hub that can communicate with many other organs [51, 52], making *Drosophila* a suitable model to study the complex integrative physiology. Some neuronal fibers terminate on the underlying epithelium, so epithelial innervation provides an access to the brain, making it plausible that α-syn spreads from cell to cell in a prion-like manner. Moreover, the midgut of adult *Drosophila* is maintained by ISCs that directly differentiate into absorptive enterocytes and secretory enteroendocrine cells. These secretory enteroendocrine cells are neural-like cells and secrete sets of hormone peptides reminiscent of neuroendocrine cells in the *Drosophila* brain [53, 54]. These enteroendocrine cells display characteristics that are typical of neurons, being best known for their membrane excitability and hormone secretion. They reside within the mucosa of the intestine in a particular anatomical orientation, allowing them to respond to signals in the lumen of the intestine. This newly recognized enterocyte-neural circuit raises many interesting possibilities. First, enterocytes may be a portal for the entry of pathogens into the nervous system [55], which aggravates the pathology of PD induced by brain α-syn. Second, enterocytes can receive bacterial stimuli from the intestine lumen, and send messages to enteric nerves, and eventually the brain, by regulating hormones or neurotransmitters [56, 57]. These findings suggest that enterocytes may be susceptible to pathogen or toxin exposure that could affect the misfolding of α-syn, which could be the first step in a prion-like cascade leading to PD. A recent study reported that enteropathogen infection in *Drosophila* modulates olfaction through metabolic reprogramming of ensheathing glia in the brain [58]. Due to the age-related intestinal inflammation, metabolic reprogramming of ensheathing glia induced by gut-derived inflammatory cytokines causes long-lasting changes in a sensory system in ageing or diseased flies. Given the potent role of glia in neurodegeneration [59], it is proposed that dysbiosis aggravates the progression of this disease through inter-organ communications. In addition, intestinal dysplasia is frequently accompanied by cell death, including apoptosis and necrosis [60, 61]. It is postulated that intestinal α-syn-induced dysplasia is coupled with cell death, which may contribute to PD-like phenotypes and pathology in *Drosophila*. Altogether, these studies provide an underlying mechanism for initiation and progression of α-syn pathology in the enteric nervous system.

Interactions between genetic and environmental factors, such as toxins, likely trigger pathogenesis by initiating α-syn oligomerization, aggregation, and propagation [62]. A growing number of studies indicate that the microbiome affects a range of animal physiology and diseases [63, 64]. Pathological alterations in microbial community composition, termed dysbiosis, result in microbial community dysfunction, which is linked to human diseases, including inflammatory bowel disease, obesity, pathogen infection, and colon cancer [65–67]. In the *Drosophila* model, we found that ectopic intestinal α-syn triggers the pathology of PD by disrupting the intestinal microbiome (Figs. 4, 5), suggesting that the intestinal α-syn contributes to the burden of PD pathologies through the DUOX–ROS–JNK pathway. The expression of DUOX was significantly higher in the midgut of *esg*^*TS*^ > *Syn* flies (Fig. 6), suggesting that the intestinal α-syn triggers the DUOX pathway. We have previously shown that knockdown of DUOX dampens oxidative innate immunity in flies [29], suggesting that DUOX silencing could ameliorate the progression of PD in *esg*^*TS*^ > *Syn* flies. Long-term exposure to pathogens can cause deleterious health outcomes, including metabolic alterations, immunological changes, and neurotoxicity, because α-syn misfolding is the key pathological mechanism underlying synucleinopathies, including PD, dementia with Lewy bodies, and multiple system atrophy [2, 68]. Individual heterogeneity in intestine microbiota may modulate resilience to synucleinopathy-associated disorders [69]. Animal models possessing microbiomes with lower taxonomic diversity are required to elucidate the underlying mechanisms by which microbes influence host traits. Recent studies on the intestine microbiome suggested that short-chain fatty acids and extracellular fibers, such as curli, generated from microbes in the gut, could affect α-syn aggregation [22]. Indeed, α-syn-overexpressing mice colonized with curli-producing bacteria display increased α-syn fibril reactivity and detergent-insoluble α-syn in the midbrain [13]. Interestingly, increased colonization and mucosal association with Enterobacteriaceae, such as *Escherichia coli*, as well as a positive association between Enterobacteriaceae abundance and disease severity, have been reported in individuals with PD compared to healthy controls [18, 19]. Studies have found synergy between dysbiosis and genetic predisposition in intestinal dysplasia [60, 68]. It is conceived that bacterial infection causes blood cell infiltration and immunity activation, which consequently aggravate PD pathologies. Moreover, previous studies have found that ageing is associated with innate immune activation in *Drosophila* brains [70, 71]. Given that the intestinal α-syn accelerates the ageing of flies, the intestine-derived α-syn might activate a systemic innate immune response that contributes to PD pathogenesis. It would be interesting to further determine if bacterial metabolites can modulate the α-syn-mediated pathology of PD. Commensal bacteria frequently antagonize pathogens through chemical inhibition and nutritional competitive exclusion. Advances in microbiome studies have the potential to contribute to new therapies for chronic human diseases, especially metabolic, immunological, and mental disorders [72]. Probiotics that are effective in inhibiting α-syn aggregation could be effective in halting the misfolding of other proteins [73, 74]. Hence, studies that are able to mechanistically disentangle the influence of microbiological communities on α-syn fibril reactivity are warranted. Identifying new specific cellular and molecular pathways that can explain the vulnerabilities of intestines to pathologic α-syn may lead to potential therapeutic interventions for PD.

## Conclusions

In this study, we found that the intestinal α-syn alone recapitulates many features of clinical PD, including a shorter lifespan, a loss of dopaminergic neurons, and progressive motor defects. Overexpression of α-syn disrupts intestinal homeostasis and accelerates the onset of intestinal ageing. Moreover, intestinal expression of α-syn induces dysbiosis that aggravates the pathology through the DUOX–ROS–JNK pathway. In addition, α-syn from both the gut and the brain synergizes to accelerate the progress of PD. Our study provides new insights into the pathophysiologic role of intestinal α-syn and may be useful for development of new therapeutic approaches for PD.

## Supplementary Information

**Additional file 1. Table S1:** Fly stocks used in the experiments.

**Additional file 2. Table S2:** Primers for qPCR analysis.

**Additional file 3. Table S3:** The list of genes with significant changes in RNA-seq analysis.

**Additional file 4. Table S4:** The list of bacteria identified on the genera by 16S rRNA analysis.

**Additional file 5.** **Fig. S1** Expression of GFP in adult brains by elav-GAL4 and esg-GAL4. **Fig. S2** Dysbiosis aggravates the pathology of Parkinson’s disease in the intestine. **Fig. S3** Intestinal α-syn triggers the DUOX-ROS-JNK pathway.

## Data Availability

The key data are included in this published article and its supplementary information files. Other datasets are available from the corresponding author upon reasonable request.

## Abbreviations

α-syn: α-Synuclein
PD: Parkinson’s disease
DA: Dopaminergic
ISCs: Intestinal stem cells
EBs: Enteroblasts
PBS: Phosphate-buffered saline
GO: Gene ontology analysis
GFP: Green fluorescent protein
TH^+^: Tyrosine hydroxylase-positive
PH3: Phospho-histone3
Dlg: Disc large
GF: Germ-free
DUOX: Dual oxidase
JNK: Jun N-terminal Kinase
