# Supplementary material for: Intestine-derived α-synuclein initiates and aggravates pathogenesis of Parkinson’s disease in Drosophila
Source: Transl Neurodegener. 2022 Oct 17;11:44. doi: 10.1186/s40035-022-00318-w (PMC9575256; doi:10.1186/s40035-022-00318-w)
Supplement: Supplementary file 1 — Additional file 1. Table S1: Fly stocks used in the experiments. [file 40035_2022_318_MOESM1_ESM.docx]

Supplementary Table 1 Fly stocks used in the experiments.

| Strains | Source | Reference number | References |
| --- | --- | --- | --- |
| *w^1118^* | Bloomington stock center | 3605 |  |
| *esg-Gal4, UAS-GFP*; *tubulin-Gal80^TS^* |  |  | (Zhai et al., 2018) |
| *w^*^;P{UASHsap\SNCA.A30P}40.1* | Bloomington stock center | 8147 |  |
| *P{hsFLP}12, y^1^ w^*^ P{αTub84B(FRT.Myc)GAL4.Bb}1; P{UAS-EGFP}5a.2* | Bloomington stock center | 64767 |  |
| *w^*^; P{UAS-bsk.B}2* | Bloomington stock center | 9310 |  |
| *ddc-gal4, UAS-**Synuclein (III)* | Temasek Life Sciences Laboratory, Singapore |  | Tong Wey Koh’s Lab, Unpublished data |
| *UAS-**Jun^bZIP^* |  |  | (Kockel et al., 1997) |
