# Supplementary material for: Intestine-derived α-synuclein initiates and aggravates pathogenesis of Parkinson’s disease in Drosophila
Source: Transl Neurodegener. 2022 Oct 17;11:44. doi: 10.1186/s40035-022-00318-w (PMC9575256; doi:10.1186/s40035-022-00318-w)
Supplement: Supplementary file 2 — Additional file 2. Table S2: Primers for qPCR analysis. [file 40035_2022_318_MOESM2_ESM.docx]

**Supplementary Table S2. Primers for qPCR analysis.**

| *Dpt F* | ACCGCAGTACCCACTCAATC |
| --- | --- |
| *Dpt R* | CCCAAGTGCTGTCCATATCC |
| *Actc F* | TTGTCTGGGCAAGAGGATCAG |
| *Actc R* | ACCACTCGCACTTGCACTTTC |
| *Drs F* | GTACTTGTTCGCCCTCTTCG |
| *Drs R* | CTTGCACACACGACGACAG |
| *Upd3 F* | GCAAGAAACGCCAAAGGA |
| *Upd3 R* | CTTGTCCGCATTGGTGGT |
| *Duox F* | CATTCCCCTGGACTCGCAC |
| *Duox R* | TCGTGCGATTGGGTGGAC |
| *Actin F* | TTGTCTGGGCAAGAGGATCAG |
| *Actin R* | ACCACTCGCACTTGCACTTTC |
| *Bacterium Universal 16s rRNA F* | TCCTACGGGAGGCAGCAGT |
| *Bacterium Universal 16s rRNA R* | GGACTACCAGGGTATCTAATCCTGTT |
| *Proteobacteria F* | CCAGGGCTTGAATGTAGAGGC |
| *Proteobacteria R* | CCTTGCGGTTCGCTCACCGGC |
| *Bacilli F* | CGACCTGAGAGGGTAATCGGC |
| *Bacilli R* | GTAGTTAGCCGTGGCTTTCTGG |
| *DECad F* | GACGAATCCATGTCGGAAAA |
| *DECad R* | TCACTGGCGCTGATAGTCAT |
| *dlg1 F* | AGAGTCGCGATGAGAAGAATG |
| *dlg1 R* | GCTGGTGCTGCTCACAACT |
| *pyd F* | TGAATCGAGAGGCAACTTCTT |
| *pyd R* | TTCTCGCGGGACAGACTC |
| *kune F* | AGGTTGTGGGCTCTGTTTTC |
| *kune F* | ATCCCGAGAATCTCCTTTGG |
