# Supplementary material for: Intestine-derived α-synuclein initiates and aggravates pathogenesis of Parkinson’s disease in Drosophila
Source: Transl Neurodegener. 2022 Oct 17;11:44. doi: 10.1186/s40035-022-00318-w (PMC9575256; doi:10.1186/s40035-022-00318-w)
Supplement: Supplementary file 5 — Additional file 5. Fig. S1 Expression of GFP in adult brains by elav-GAL4 and esg-GAL4. Fig. S2 Dysbiosis aggravates the pathology of Parkinson’s disease in the intestine. Fig. S3 Intestinal α-syn triggers the DUOX-ROS-JNK pathway. [file 40035_2022_318_MOESM5_ESM.pdf]

Supplementary Fig. 1

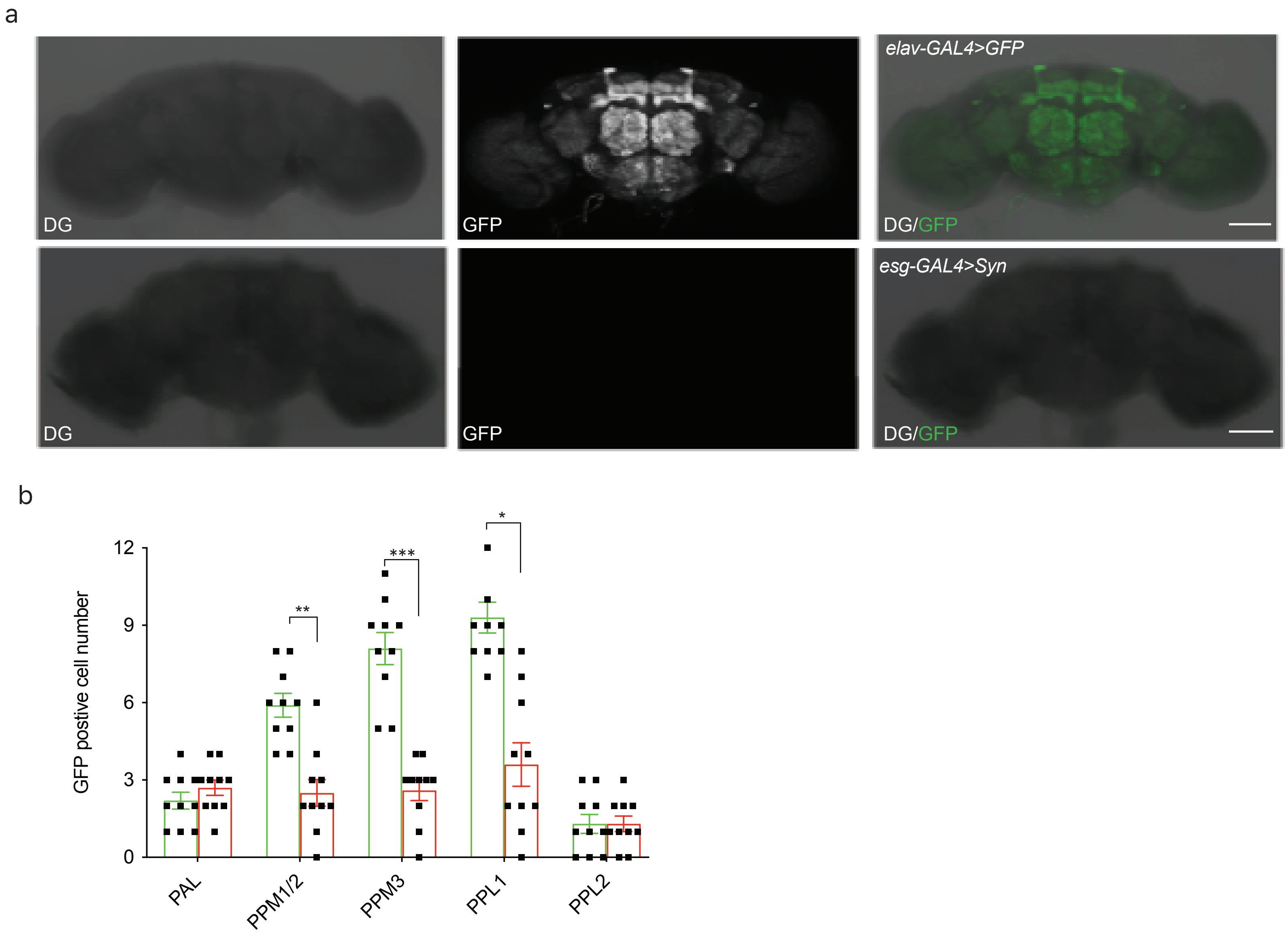

Fig. S1 Expression of GFP in adult brains by *elav-GAL4* and *esg-GAL4*. **a** Temperature-controlled expression of *Syn* and *GFP* was achieved by co-expression of *esg-GAL4* with a temperature-sensitive version of the GAL4 inhibitor, Gal80^{TS} as described in Fig. 1a. Green: GFP; DG: dark-ground. Scale bar: 50 μm. **b** The number of GFP-positive cells labeled by TH-GAL4 in 28-day old flies. Mean ± SEM; the *p*-values of survival curves were calculated using log-rank tests (using total fly numbers), and for category graphs using a one-way ANOVA with a Bonferroni multiple-comparison test. *$P < 0.05$; **$P < 0.01$; ***$P < 0.001$.

Supplementary Fig. 2

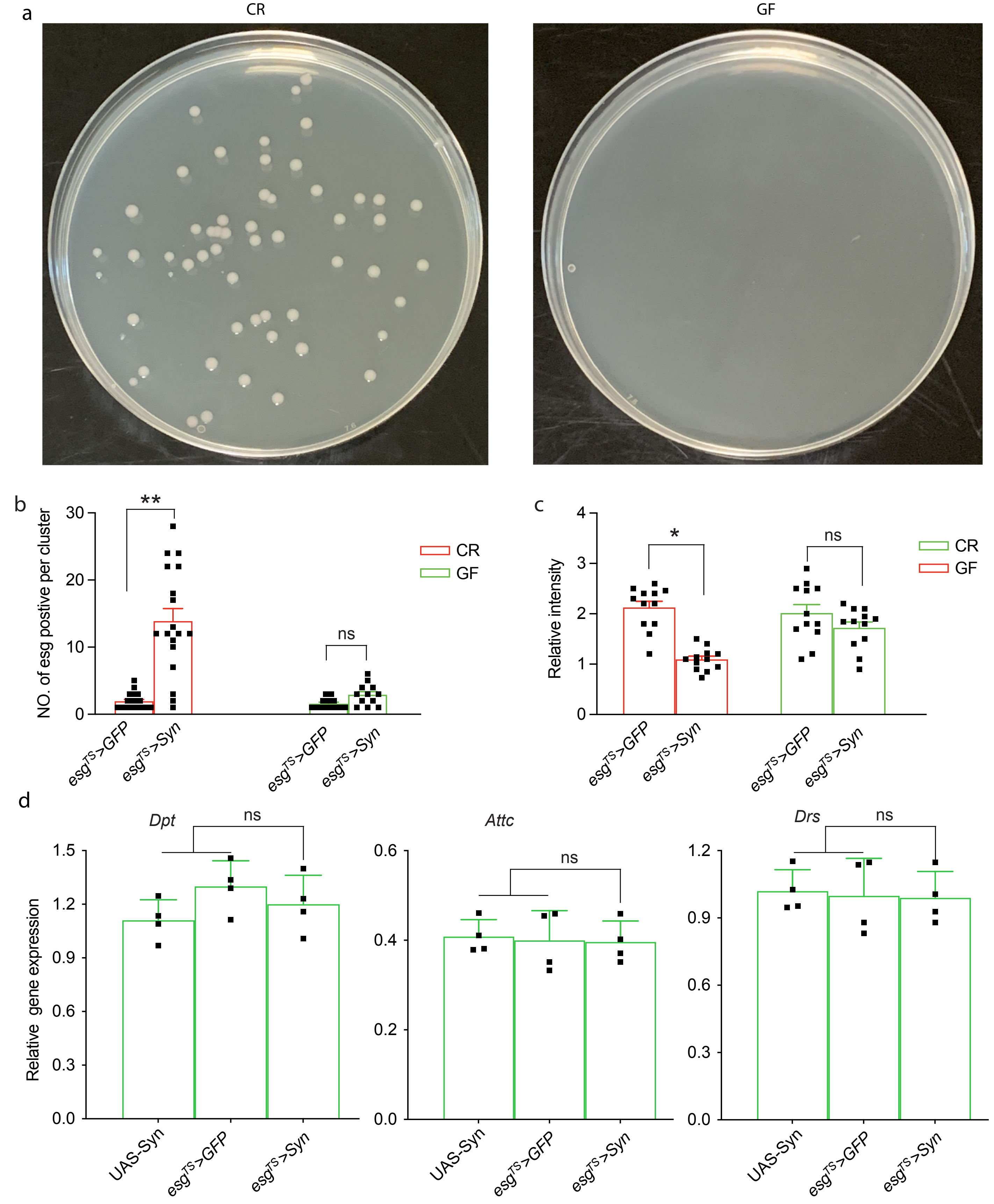

Fig. S2 Dysbiosis aggravates the pathology of Parkinson's disease in the intestine. **a** Verification of axenia of GF flies. Bacterial load was determined by plating the homogenate of fly midguts with 1: 1,000 dilution on LB agar plates. Representative images are shown. CR: conventionally reared; GF: germ free. **b** The quantification of cell numbers in esg-positive cell clusters in the anterior midgut. **c** Fluorescence intensity of anti-Dlg signals in intestinal cells. d Activation of intestinal immunity by intestinal α-syn was dampened by antibiotics cocktail. Antimicrobial peptide gene expression of middle intestines of 28-day-old *Drosophila* using qPCR. *Dpt*, *Diptericin*; *Attc*, *Attacin C*; *Drs*, *Drosocin*. $n = 4$.

Supplementary Fig. 3

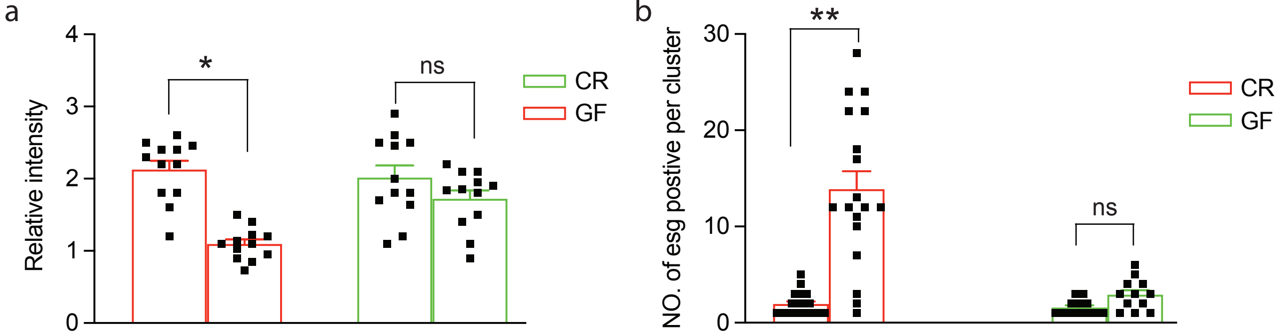

Fig. S3 Intestinal α-syn triggers the DUOX-ROS-JNK pathway. **a** Fluorescence intensity of anti-p-JNK signals in intestinal cells was calculated. $n = 60$ for each. **b** The percentage of ISC and enterocytes (EC) in total p-JNK-positive cells. $n = 236$.
